# Hepatic Visceral Larva Migrans of *Toxocara canis*

**DOI:** 10.4269/ajtmh.2010.09-0602

**Published:** 2010-04

**Authors:** Jae Hoon Lim

**Affiliations:** Department of Radiology and Center for Imaging Science, Sungkyunkwan University School of Medicine, 50 Ilwond-dong, Kangnam-gu, Seoul 135-710, Korea

**Figure 1.**
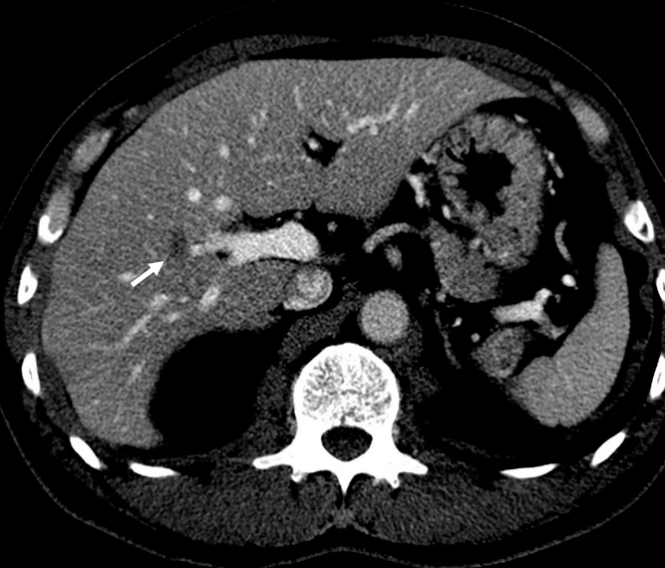
Contrast-enhanced CT shows a small, oval, low-attenuating nodule in the right lobe of the liver adjacent to the anterior branch of the right portal vein (arrow).

**Figure 2.**
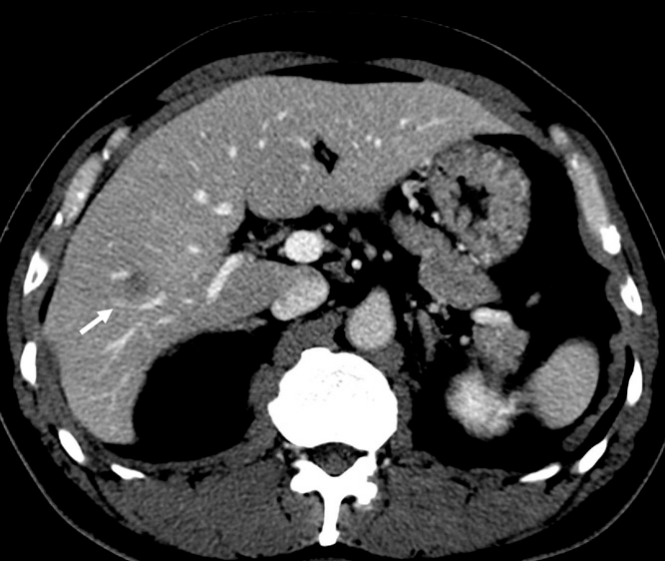
The CT image obtained 3 months later shows that the nodule migrates slowly upward and anteriorly in the liver (arrows).

**Figure 3.**
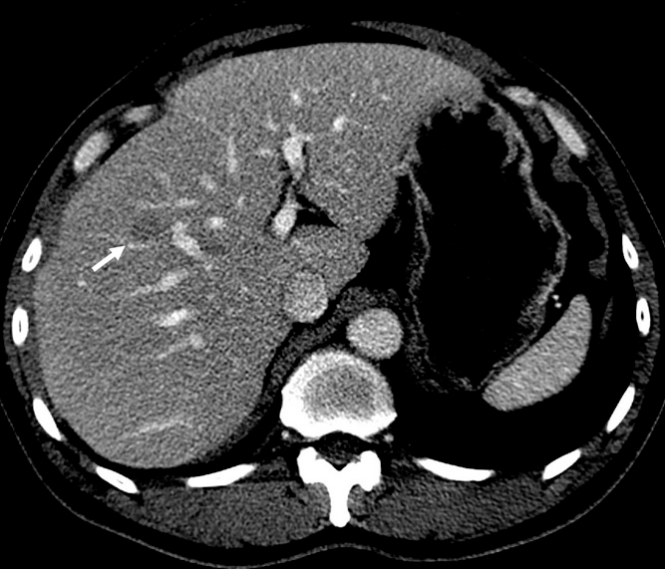
The CT image obtained 6 months later shows that the nodule migrates slowly upward and anteriorly in the liver (arrows).

**Figure 4.**
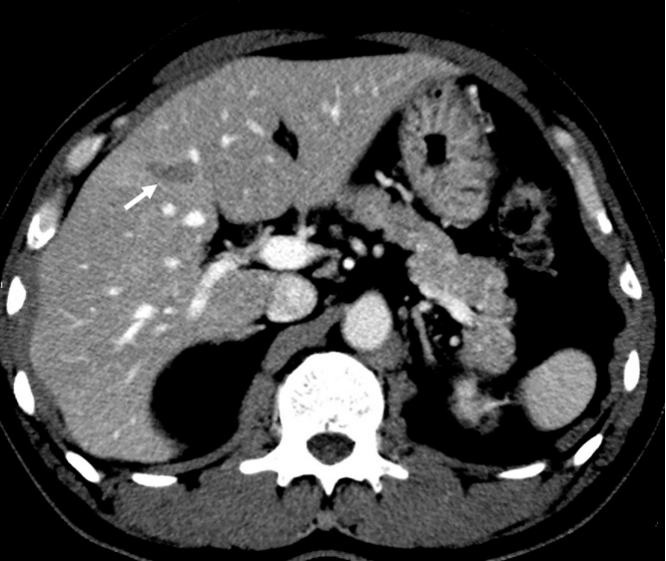
The CT image obtained 10 months later shows that the nodule migrates slowly upward and anteriorly in the liver (arrows).

**Figure 5.**
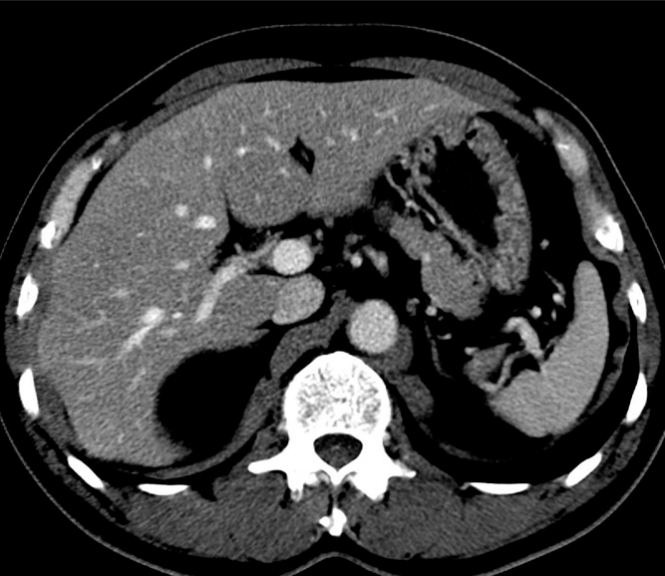
The CT image obtained 15 months later discloses no nodule.

A 54-year-old man with diabetes mellitus had a blood test and was found to have a blood eosinophilia (1,028/mm^3^). Liver-function tests were normal. A hepatic nodule was detected on screening of upper abdominal and renal sonography. Contrast-enhanced computed tomography (CT) scan (Figure 1) showed a small, oval, low-attenuating nodule in the right hepatic lobe. CT scans obtained 3, 6, and 10 months later (Figures 2–4) revealed that the nodule was migrating. During this 10-month time period, eosinophilia persisted (720–1,821/mm^3^). A CT scan obtained 15 months later (Figure 5) revealed the disappearance of the nodule, and the eosinophil count returned to normal (300/mm^3^). An enzyme-linked immunosorbent assay (ELISA) for *Toxocara canis* was strongly positive, and optical density was 2.53 (cut-off value = 0.69). He had several occasions of eating chops of uncooked cow's liver at restaurants before the initial blood test.

As in dogs and animals, human infection of *T. canis* takes place in two ways^1^–^3^—by ingestion of embryonated eggs or alternatively, by transfer of the arrested larvae in the tissues of a paratenic host to humans. According to reports, the prevalence rate of intestinal toxocariasis in dogs was 18.9%,^4^ and larva recovery rate from beef liver was 11.8%.^5^ In certain ethnic groups, some adults tend to eat uncooked chops of cow's liver that contain arrested infective larvae. After swallowing, these arrested larvae are released in the intestine during the digestion process and then, they pass through the intestinal wall, get into the portal vein, reach the liver, and move slowly, exhibiting larva migrans.^6^
